# An enclosed rotating floating photobioreactor (RFP) powered by flowing water for mass cultivation of photosynthetic microalgae

**DOI:** 10.1186/s13068-016-0633-8

**Published:** 2016-10-18

**Authors:** Jim Junhui Huang, Gagarin Bunjamin, Edwin Sianguan Teo, Deric Boonhuat Ng, Yuan Kun Lee

**Affiliations:** 1 Singapore Peking University Research Centre for a Sustainable Low-Carbon Future (SPURc), CREATE Tower, 1 Create Way, Singapore, 138602 Republic of Singapore; 2 Department of Microbiology and Immunology, National University of Singapore, 5 Science Drive 2, Singapore, 117597 Republic of Singapore; 3 Algae Enviro-Engineering Pte Ltd, 18 Gul Avenue, Singapore, 629660 Republic of Singapore

**Keywords:** Rotating floating photobioreactor, *Dunaliella tertiolecta*, Sodium bicarbonate, Biomass productivity, Metabolites, Biofuels

## Abstract

**Background:**

The design of photobioreactor (PBR) for outdoor mass cultivation of microalgae determines the distribution of solar irradiance among cells in the culture, mode of agitation, mass transfer efficacy, and energy consumption, thus determines the productivity of the system and the cost of production. In this study, the concept of a floating photobioreactor with rotation function is proposed. *Dunaliella tertiolecta*, a model microalga, cultured in the attached vessels was evaluated.

**Results:**

The rotation of the photobioreactor was powered by flowing water, in this case waves generated through a paddle wheel in an outdoor raceway pond for proof of concept. The rotating floating PBR (RFP) could be powered by natural flowing stream, river, and tidal waves, thus there could be no energy cost for agitation of the cultures in maintaining the cells in suspension. This RFP is characterized by its energy-saving and temperature control properties as well as more homogenous light distribution in the culture as compared to conventional culture systems, such as raceway pond. Maximal cell concentration of 8.38 × 10^6^ cells mL^−1^, biomass productivity of 3.10 g m^−2^ day^−1^, and photosynthetic efficiency of 4.61 % (PAR) were achieved. In addition, satisfactory productivities of *D. tertiolecta* metabolites including carotenoids, mycosporine-like amino acids and lipids were also obtained.

**Conclusions:**

The RFP, powered by flowing water, creates an innovative culture technology for economical cultivation of microalgal cells and production of microalgal metabolites.

## Background

The excessive dependence on fossil fuels since industrial revolution might eventually evolve into energy crisis that was a seriously social problem which would cause economic recession, unemployment, social instability, and even lead to war [1]. For solving such issue, various new bioenergy forms such as bio-hydrogen generated by biophotolysis and photo/dark fermentation, bio-ethanol from food crops like corn grain and sugar cane, bio-methane using agricultural residue and wastes from agro-industries, along with oil-seed-crop-based biodiesels derived from *Jatropha curcas* and palm termed the first and secondary generations of biofuels have been proposed and developed for decades. However, these approaches involve complicated purification technologies, competition with food provision and low productivities of oil plants [2–5]. Microalgae are recognized as potential source of bioenergy and regarded the third generation of biofuel [6].

Photobioreactors (PBRs) for microalgal cultures were rapidly developed in recent years [7]. Mass cultivation of microalgae could be classified into open and closed types. The open types include circular and raceway ponds for cultivation of fast growing microalgae such as *Chlorella,* and microalgae that tolerate extreme environments such as *Spirulina* to high salinity and alkalinity [8]. On the other hand, there are enclosed PBRs consisting of flat plate or tubing with different configurations, for instance, vertical, horizontal, and inclined that could be suitable for the cultivation of microalgae sensitive to environmental conditions and liable to contamination by environmental microbes [9–11]. Whichever the designs, cost of energy to facilitate mass transfer and maintaining algal cells in suspension contribute to as much as 31 % of the production cost based on the most cost-effective tubular photobioreactor, leading to high cost of algal biomass and products [12].

Water-based floating PBRs free the use of lands and maintain culture temperature [13, 14]. Water movement in the seas provides agitation to bag-type PBR [15].

In this study, a conceptual model of rotating floating PBR (RFP) is proposed, designed, constructed, and evaluated in a flowing pond. *Dunaliella tertiolecta* (Chlorophyceae), a model marine green microalga widely studied in the laboratory and commercial outdoor cultivation, was selected as the tested organism due to its fast-growth rate, high CO_2_ sequestration, and rich in β-carotene [16].

## Methods

### Rotating floating photobioreactor

**Fig. 1:**
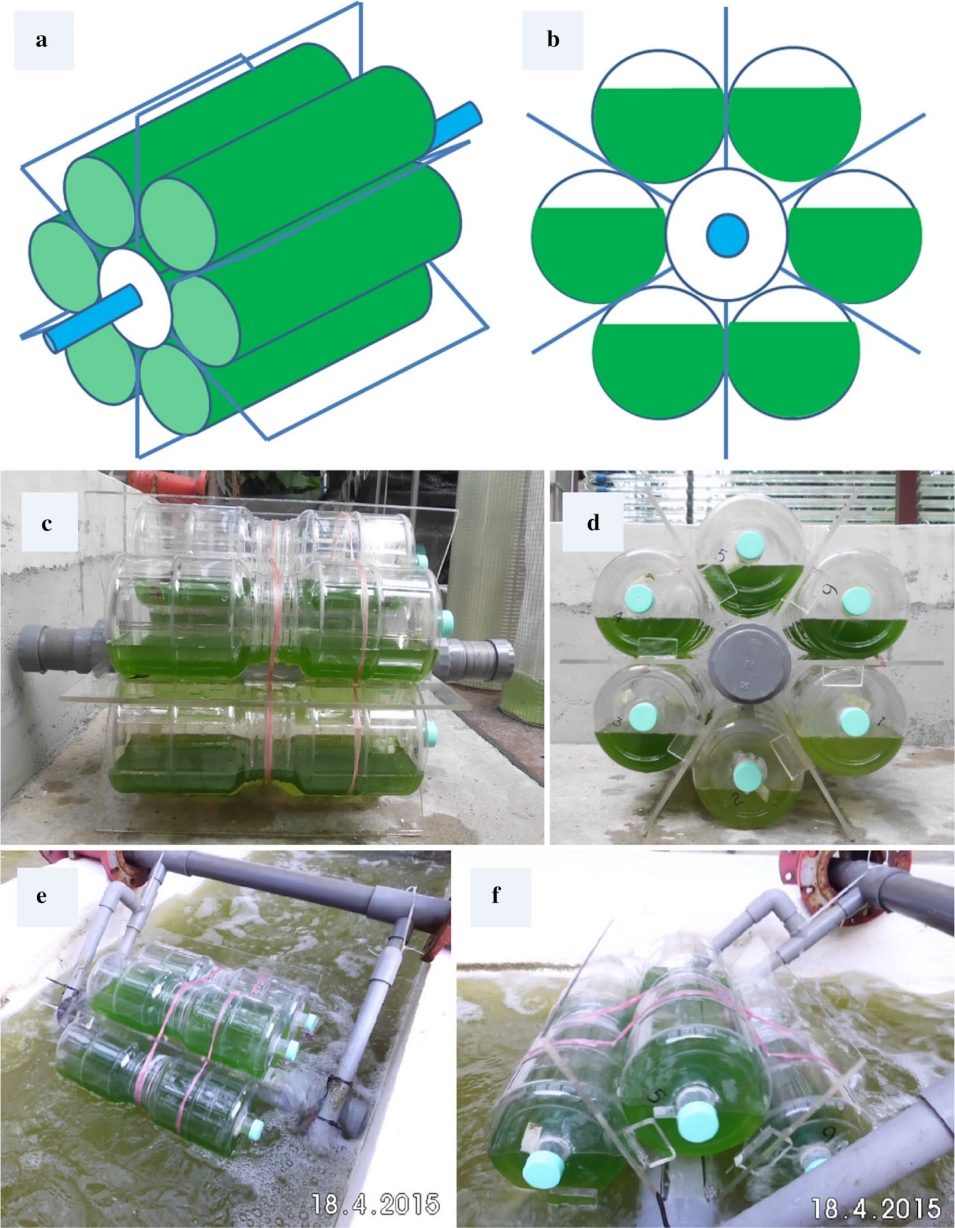
The schematic diagrams of rotating floating photobioreactor. **a** The 3D schematic diagram of the rotating floating PBR. **b** The cross section of schematic diagram of rotating floating PBR. **c** The *front view* of rotating floating PBR filled with culture. **d** The *cross-section view* of rotating floating PBR filled with culture. **e** A rotating floating PBR placed in a raceway pond viewed from *top*. **f** A rotating floating PBR placed in a raceway pond viewed from side

The conceptual model of RFP (Fig. 1c, d) was designed and constructed according to the schematic diagrams shown in Fig. 1a, b. The main structure of this RFP is composed of an axis, 9 cm in diameter, made of PVC plastic, and six pieces of Plexiglas with 6 mm thickness. The Plexiglas served as paddles for pushing and rotating the RFP around the axis, by means of unidirectional wave. Six 5 L barrels made of PET transparent plastic filled the spaces between the paddles. An outlet at the flank of every barrel was inserted to permit sampling and release of gas. The size of RFP was 74 cm in length and 47 cm in width to jointly form a water footprint of 0.3478 m^2^ for each RFP. Each of the 5-L barrels held 3-L of culture medium to generate enough buoyancy for RFP to float on the water. The illuminated surface area was estimated to be 0.1558 m^2^ (41 cm in length and 38 cm in width, culture surface area). The axle of the RFP was attached to two long orbit shafts fixed at the wall of the pond, allowing the RFP paddles to move clockwise and reach an average rotating speed of 3 s per circle.

### Microalgae strains

*Dunaliella tertiolecta* strain LB-999 was obtained from UTEX Culture Collection of Algae (University of Texas at Austin, TX, USA). *D. tertiolecta* cells were maintained in sterile ATCC-1174 DA liquid medium (American Type Culture Collection at Manassas, Virginia, USA) containing 0.5 M NaCl in Erlenmeyer flasks at 25 °C under a light:dark regime of 12:12.

### Cultivation conditions

Freshly prepared bicarbonate-free ATCC-1174 DA liquid medium (2.0 M NaCl) was sterilized and 950 mL of which was inoculated with 50 mL of the alga to give a cell concentration of 2.89 × 10^5^ cells mL^−1^. In order to compare the influences of different sodium bicarbonate levels on the growth of *D. tertiolecta*, six RFP barrels sterilized by ethanol were divided into three groups: the control (bicarbonate-free), Treatment 1 (1.68 g L^−1^ sodium bicarbonate), and Treatment 2 (8.40 g L^−1^ sodium bicarbonate). This outdoor experiment was carried out during April 12–30, 2015, at 18 Gul Avenue, Singapore 629660 (1°19′N, 103°68′E), Republic of Singapore. The RFP was illuminated with solar irradiation. Photosynthetically active radiation (PAR) intensities were measured by a PAR quantum sensor (Skye Instruments Ltd., UK). The temperatures of pool water and culture in RFP were determined daily by means of a sterile thermometer, respectively.

### Determination of biomass and related parameters

Fifteen mL *D. tertiolecta* cultures were sampled. Before biomass determination, the pH values of these culture samples were measured using a Mettler-Toledo AG pH meter (Mettler-Toledo International Inc., Switzerland). For cell concentration and cell size determination, 50 µL of Lugol’s solution was added to 950 µL of each culture sample and measured by a Dual fluorescence cell counter (Luna™ *fL*, Korea). *D. tertiolecta* biomass was monitored using optical density (at wavelength 685 nm) and cell-free culture medium as the control in an Agilent Cary 60 UV–Vis spectrophotometer (Agilent Technologies, USA). The dry weight (DW) was verified by drying 20 mL of culture on pre-weighed (*m*_1_) glass microfiber filters (Whatman®, 47 nm, nominal pore size 0.7 µm, UK). Culture supernatant was firstly removed by vacuum filtration between 35 and 55 mmHg, washed by 20 mL 0.5 M ammonium bicarbonate, and lyophilized in a freeze-drier (Freeze dry/Shell freeze system, LABCONCO, USA) to reach a constant weight. Finally, the dried filter was weighed (*m*_2_), and DW of *D. tertiolecta* was calculated as follows [17]:

1 $${\text{Dry weight }}\left( {{\text{g L}}^{ - 1} } \right) \, = \frac{{m_{2} \,({\text{g}}) - m_{1} \,({\text{g}})}}{{0.02\,({\text{L}})}}.$$

The specific growth rate (*μ*) was calculated by the equation below:

2 $$\mu \,\left( {{\text{h}}^{ - 1} } \right) \, = \frac{{Ln N_{n} - Ln N_{0} }}{{24n\,({\text{h}}^{ - 1} )}},$$

where *N*_n_ and *N*_0_ stand for the cell numbers per mL of culture measured on Day *n* and Day 0 at the same hour, respectively, and *n* represents the time of cultivation (days) [17].

The doubling time (*t*_d_) was calculated according to its relation with *μ* as follows:

3 $$t_{\text{d}} \left( {\text{h}} \right) \, = \frac{0.693}{{\mu ({\text{h}}^{ - 1} )}}.$$

### Determination of productivity

As mentioned in “Rotating floating photobioreactor” section, the water footprint for one RFP was 0.3478 m^2^ and a RFP might contain six culture barrels that total 18 L of culture, thus 1 m^2^ of footprint of RFP contained 51.75 L of culture. The productivities in this study included those of biomass, total carotenoids, total mycosporine-like amino acids (MAAs) and total lipids and were calculated as follows:

4 $${\text{Productivity}}\left( {{\text{g m}}^{ - 2} \, {\text{day}}^{ - 1} } \right) \, = \frac{{M_{n} - M_{0} }}{n},$$

where the *M*_n_ and *M*_0_ stand for the mass (g m^−2^) measured on Day *n* and Day 0, respectively, and *n* represents the time of cultivation (days) [18].

### Determination of photosynthetic efficiency

The photosynthetic efficiency (PE) was defined as follows:

5 $${\text{PE}}\left( \% \right) \, = \frac{{E_{B} }}{{E_{L} }} \times 100\,\%,$$

in which the *E*_*B*_ and *E*_*L*_ stand for the free energy included in biomass and in light energy (PAR 400–700 nm), respectively. PE value was calculated based on the following assumption: (1) On the average, 1 mol of visible photon contains 217 kJ energy; (2) under normal growth condition without stress, 1 g of algal biomass contains 20 kJ energy [18].

### Determinations of pigments

Chlorophyll *a*, chlorophyll *b*, and total carotenoid content were determined. The freeze-dried filters mentioned in “Determination of biomass and related parameters” section containing dried *D. tertiolecta* cells were cut into small pieces and grounded in a mortar containing 1.6 mL of 100 % acetone (HPLC grade, Sigma, USA) for 5 min in the dark. The acetone extract was kept in an ice bath, while 1.6 mL of 100 % acetone was used to wash the mortar and added to the initial extract. Then 0.8 mL of Milli-RO water was added to produce a final acetone concentration of 80 %. The extract was sonicated (Ultrasonic Cleaner ALD-40050-07, ALSTRON™, Singapore) for 10 min, to fully extract the pigments from cell debris. The extracts were centrifuged at 3500*g* (2–16, Sigma®, Sartorius, Germany) for 10 min, the supernatant was used for pigment determination.

Determination of chlorophyll *a*, chlorophyll *b*, and total carotenoids contained in the *D. tertiolecta* cells was adapted from Yang and colleagues [19]. The absorbance of the supernatant was measured at the wavelengths of 663.6, 646.6, and 440.5 nm by an Agilent Cary 60 UV–Vis spectrophotometer (Agilent Technologies, USA). The contents of chlorophyll *a*, chlorophyll *b,* and total carotenoids were calculated by the following formula on the basis of mg gDW^−1^:

6 $$\begin{aligned}&{\text{Chlorophyll}}\;a\;{\text{content}}\,\left( {{\text{mg gDW}}^{ - 1} } \right) \\ &\quad= \frac{{(12.25A_{663.6} - 2.55A_{646.6} )\,({\text{mg L}}^{ - 1} )}}{{{\text{Dry weight}}\,({\text{gDW L}}^{ - 1} )}}\end{aligned}$$

7 $$\begin{aligned}&{\text{Chlorophyll}}\;b\;{\text{content}}\,\left( {{\text{mg gDW}}^{ - 1} } \right) \, \\ &\quad= \frac{{(20.31A_{646.6} - 4.91A_{663.6} )\,({\text{mg L}}^{ - 1} )}}{{{\text{Dry weight}}\,({\text{gDW L}}^{ - 1} )}}\end{aligned}$$

8 $$\begin{aligned}&{\text{Total}}\;{\text{carotenoids}}\;{\text{content}}\,\left( {{\text{mg gDW}}^{ - 1} } \right) \, \\ &\quad= \frac{{(4.69A_{440.5} - 4.74A_{646.6} - 1.96A_{663.6} )\,({\text{mg L}}^{ - 1} )}}{{{\text{Dry weight}}\,({\text{gDW L}}^{ - 1} )}}.\end{aligned}$$

### Determination of total mycosporine-like amino acids (MAAs)

The determination on total UV-absorbing MAAs was based on protocols of Dunlap et al. with some modifications [20]. An 80 % acetone extract of *D. tertiolecta* mentioned in “Determinations of pigments” section was prepared and its absorption spectrum was scanned from 300 to 750 nm using an Agilent Cary 60 UV–Vis spectrophotometer (Agilent Technologies, USA). The total MAA content was calculated by the following formula on the basis of mg gDW^−1^, in which *A*_MAAs_ refers to total peak area in the range of 319–350 nm, and *A*_Chl*a*_ stands for peak area range 630–680 nm as shown in Fig. 5f. In this study, OriginPro 8 software was used to calculate the values of *A*_MAAs_ and *A*_Chl*a*_.

9 $$\begin{aligned}&{\text{Total}}\;{\text{MAAs}}\;{\text{content}}\,\left( {{\text{mg gDW}}^{ - 1} } \right) \, \\ &\quad= \frac{{(12.25A_{663.6} - 2.55A_{646.6} )\,({\text{mg L}}^{ - 1} )}}{{{\text{Dry weight}}\,({\text{gDW L}}^{ - 1} )}} \times \frac{{A_{\text{MAAs}} }}{{A_{{{\text{Chl}}a}} }} .\end{aligned}$$

### Determination of crude lipids and total lipids

*D. tertiolecta* culture samples were collected on Days 0, 3, 6, 9, 12, 15, and 18 for lipid measurement. The culture samples were centrifuged at 3500*g* (2–16, Sigma®, Sartorius, Germany) for 10 min. The supernatants were discarded and the pellets were washed by Milli-RO water twice to remove salt attached on the surface of *D. tertiolecta* cells. Finally, the pellets were lyophilized in freeze-drier (Freeze dry/Shell freeze system, LABCONCO, USA) to constant weight. Determination of *D. tertiolecta* crude lipids followed the Bligh–Dyer method [17]. 50–100 mg of the above-mentioned dried *D. tertiolecta* sample (M) was put into a pre-weighed (*m*_1_) 5 mL glass vial with a PTFE cap followed by adding 0.4 mL of Milli-RO water, 1.5 mL of CHCl_3_/MeOH (1:2, v/v) and was vigorously vortexed for 5 min. Afterwards, 0.5 mL of CHCl_3_ was added and the vial was vortexed again for another 5 min. Subsequently, 0.5 mL of Milli-RO water was added and the mixture was vortexed for the last 5 min. After that, the vial was centrifuged at 3500*g* (2–6, Sigma®, Sartorius, Germany) for 10 min to produce an upper aqueous layer and a lower organic layer. The organic layer was then transferred to a new vial and the solvent was evaporated using a nitrogen evaporator. At the end, this vial with extracted crude lipids was dried in a freeze-drier (Freeze dry/Shell freeze system, LABCONCO, USA) to reach a constant weight (*m*_2_). Crude lipid content of *D. tertiolecta* was calculated as follows:

10 $$\begin{aligned}&{\text{Crude}}\;{\text{lipids}}\;{\text{content}} \, \left( {{\text{mg gDW}}^{ - 1} } \right) \, \\ &\quad= \frac{{m_{2} ({\text{mg}}) - m_{1} ({\text{mg}})}}{{M ({\text{gDW}})}}.\end{aligned}$$

As all the pigments (chlorophyll and carotenoids) were extracted at the same time, the total lipid level in *D. tertiolecta* was estimated by subtracting the concentrations of chlorophyll *a*, chlorophyll *b,* and total carotenoids calculated in formula (6), (7), and (8) from the crude lipid levels determined in formula (10).

### Statistical analysis

All experiments were performed and repeated in the identical RFP, the results were analyzed by one-way analysis of variance (ANOVA). Post-hoc analyses were made by Tukey’s multiple comparison or Student’s *t* test to estimate the differences between the control and treatments. Differences were considered significant at *p* < 0.05. All statistics were performed with SigmaStat (version 3.1) software (SPSS).

## Results and discussion

To the best of our knowledge, our invention is the first to design and construct a floating photobioreactor rotating on a water body by means of wave power, in providing agitation and to facilitate distribution of solar irradiance across the culture system. There are several advantages on this RFP, which include energy saving, temperature control, better light distribution, and design simplicity that should be highlighted as compared to traditional land-based photobioreactors and other floating photobioreactors [13, 14].

### RFP evaluation

Different from land-based PBR that required high (electrical mostly) energy input to provide mixing in the cultures, RFP which is energy saving (thus reduce operation cost) for the culture system is moved by natural wave powers such as river, stream, and sea wave. It is conceivable that the mixing in RFP is better than other reported floating PBRs because the rotation is unidirectional and achieving upside down rotation of the culture. The second benefit of RFP lies in its temperature control, which is often difficult to achieve in land-based large-scale outdoor PBR. For the RFP, part of the culture is submerged and rotating in water, which serves to prevent heating of the culture, as demonstrated in this study. In this study, the culture temperature was maintained at around 25 °C at night and 29 °C at noon.

The third and most important advantage of RFP is light distribution, which is often the bottleneck in most algal culture systems. The rotation movement caused the cultures in the six culture barrels to move in and out of water level periodically. This ensures exposure of all photosynthetic cells to solar irradiance and at the same time dissipates heat of solar irradiance captured by the culture.

Nevertheless, in any PBR, a fraction of the culture might receive light intensity lower than the critical level for photosynthesis due to self-shading and reactor design. A study has demonstrated that *Chlorella* culture could continue to grow at its maximum specific growth rate in the dark for 9.2 s in a light saturate culture [21]. The rotating rate of the RFP was only 3 s per cycle, implying that ATP and NADPH accumulated in the light was probably able to maintain growth of the culture in the dark portion of the culture system to sustain continuous growth.

The last merit of RFP came from the simplicity of its design. It does not need complicated system and equipment for CO_2_ provision and for O_2_ removal as in conventional PBRs [22].

**Fig. 2:**
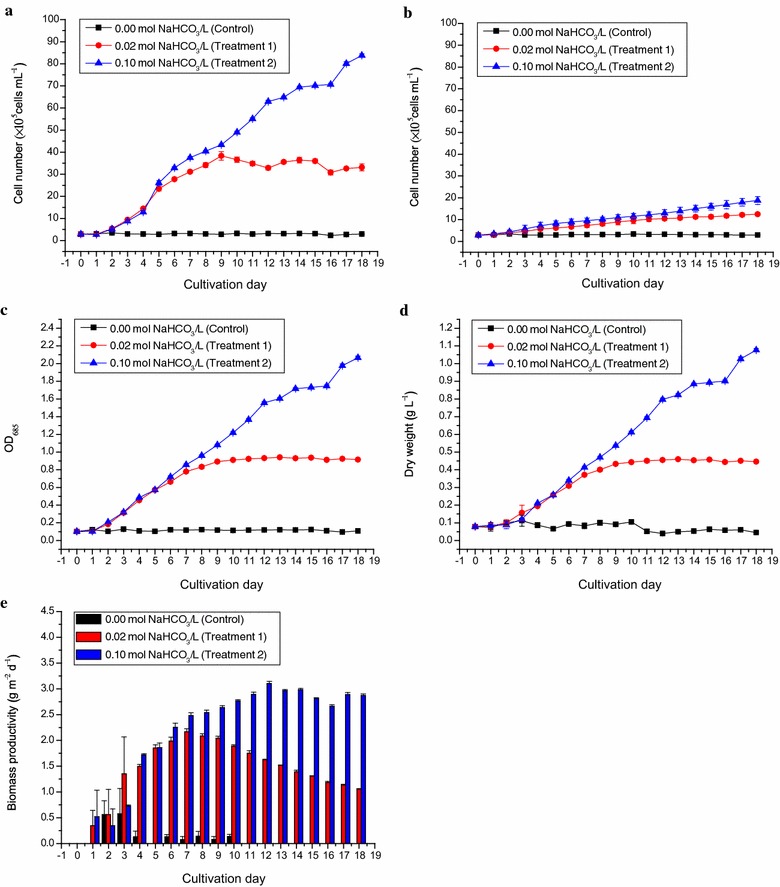
The growth curves and biomass productivities of *Dunaliella tertiolecta* cultured outdoors under different sodium bicarbonate concentrations in ATCC-1174 DA medium using rotating floating photobioreactor. **a** The changes in cell count per mL culture of *D. tertiolecta* under 0.02 mol L^−1^ NaHCO_3_ (*filled red circle*) and 0.10 mol L^−1^ NaHCO_3_ (*filled blue triangle*) as compared to the control, 0.00 mol L^−1^ NaHCO_3_ (*filled black cube*). **b** The changes in cell count per mL culture of *D. tertiolecta* in RFP without rotation/static RFP under cultivation conditions mentioned above. **c** The changes in OD_685_ values of *D. tertiolecta* culture under cultivation conditions mentioned above. **d** The changes in dry weight per liter of *D. tertiolecta* culture under cultivation conditions mentioned above. **e** The changes in biomass productivities (g m^−2^ day^−1^) of *D. tertiolecta* culture under 0.02 mol L^−1^ NaHCO_3_ (*red bar*) and 0.10 mol L^−1^ NaHCO_3_ (*blue bar*) as compared to the control, 0.00 mol L^−1^ NaHCO_3_ (*black bar*). The negative values of biomass productivities calculated were all shown as 0 g m^−2^ day^−1^. Data were expressed as mean ± standard deviation of three independent experiments (*n* = 3)

In the rotating floating-PBR culture system, the inclusion of bicarbonate as the carbon source showed a dose-dependent support of the growth of *D. tertiolecta* as shown in Fig. 2. In the control culture, without the supply of bicarbonate and CO_2_, growth parameters including cell numbers, OD_685_ value and dry weight of *D. tertiolecta* were not significantly changed (*p* > 0.05) over the 18-day cultivation (Fig. 2a, c, d). In Treatment 1 where 1.68 g L^−1^ (0.02 mol L^−1^) sodium bicarbonate was provided, 3.83 × 10^6^ cells mL^−1^ was counted on Day 9, OD_685_ of 0.94 and dry weight of 0.46 g L^−1^ were measured on Day 13. In Treatment 2 where 8.40 g L^−1^ (0.10 mol L^−1^) bicarbonate was provided, 8.38 × 10^6^ cells mL^−1^, OD_685_ of 2.07 and 1.08 g L^−1^ dry weight were measured on Day 18, respectively. These showed a 13.25-fold increased over that of the control in Treatment 1, and 28.99-fold increase over that of the control in Treatment 2 (Fig. 2a, c, d).

The biomass productivities of *D. tertiolecta* under these three bicarbonate conditions are shown in Fig. 2e. Under the control condition, there was no increase in biomass over the whole study period (Fig. 2e). However, in Treatment 1 and Treatment 2 the biomass productivities in the first 5 days were comparable. The biomass productivity of Treatment 1 reached its maximum of 2.17 g m^−2^ day^−1^ on Day 7, whereas the biomass productivity in Treatment 2 continued to increase and reached the maximum of 3.10 g m^−2^ day^−1^ on Day 12, which represented a 1.43-fold productivity of Treatment 1 (Fig. 2e). After the maxima of biomass productivities were achieved, the productivities of both treatments progressively decreased.

As shown in the results, higher (0.10 mol L^−1^) sodium bicarbonate concentration seemed to be able to support higher growth of *D. tertiolecta* in RFP (Fig. 2a), but did not resulted in proportional level of biomass which suggests that the ATCC basal medium was carbon limited, whereas in high carbonate medium other growth nutrients such as nitrogen, phosphorus, or trace metal elements became growth limiting in the *D. tertiolecta* culture (Fig. 2d).

Mixing of culture in microalgal cultivation is a critical procedure to ensure mass transfer and light irradiance distribution. This is evident from the comparison of the growth of *Dunaliella* in RFP with and without rotation over an 18-day cultivation, as shown in Fig. 2a, b. *Dunaliella* in rotating RFP reached 8.38 × 10^6^ cells mL^−1^ of cell concentration in high carbonate medium, whereas in the statically placed RFP without rotation its maximal cell concentration was only 1.87 × 10^6^ cells mL^−1^, and most of the cells were settled at the bottom of culture, suggesting that rotation was an essential motion to ensure mass transfer and solar irradiance distribution (Fig. 2a, b).

In comparison with other studies on outdoor cultivation of *Dunaliella* in PBRs, the highest biomass productivity achieved in the RFP (3.10 g m^−2^ day^−1^) is higher than that reported in a closed tubular PBR (2.20 g m^−2^ day^−1^) [16]. In a recent published report on long-term outdoor cultivation of *D. tertiolecta* in hanging bag PBRs supplied with air, the maximal cell density of approximately 1.30 × 10^6^ cells mL^−1^ and *μ* of 0.0108 h^−1^ (0.26 day^−1^) were recorded [23], both of which were significantly (*p* < 0.05) lower than our data as shown in Table 1 and Fig. 2a.

**Table 1:** Photosynthetic efficiency, specific growth rate, and doubling time of *Dunaliella tertiolecta* under different sodium bicarbonate concentrations during cultivation using rotating floating photobioreactor

Day	TPAR^a^ (mol m^−2^ day^−1^)	APARE^b^ (kJ m^−2^)	Photosynthetic efficiency (PE)			Specific growth rate (*μ*)			Doubling time (*t*_d_)		
			Control^c^ (%)	Treatment 1^d^ (%)	Treatment 2^e^ (%)	Control (h^−1^)	Treatment 1 (h^−1^)	Treatment 2 (h^−1^)	Control (h)	Treatment 1 (h)	Treatment 2 (h)
1	12.25	2659.24	0^f^	0	0	0	0	0	∞^g^	∞	∞
2	13.03	5485.94	0.82 ± 0.39	0.82 ± 0.71	0	0.0039 ± 0.0033^A^	0.0131 ± 0.0015^B^	0.0124 ± 0.0024^B^	177.69 ± 9.29^X^	53.28 ± 6.42^Y^	57.47 ± 12.30^Y^
3	11.96	8082.30	0.85 ± 0.73	2.01 ± 1.07	1.08 ± 0.03	0.0004 ± 0.0003^A^	0.0164 ± 0.0019^B^	0.0155 ± 0.0019^B^	1733.32 ± 4.35^X^	42.70 ± 5.10^Y^	45.32 ± 5.91^Y^
4	15.70	11,488.86	0.15 ± 0.08^α^	2.08 ± 0.05^β^	2.39 ± 0.03^β^	0.0005 ± 0.0004^A^	0.0168 ± 0.0010^B^	0.0156 ± 0.0014^B^	1386.78 ± 8.15^X^	41.39 ± 2.59^Y^	44.66 ± 4.27^Y^
5	9.34	13,516.02	0^α^	2.74 ± 0.10^β^	2.76 ± 0.13^β^	0^A^	0.0175 ± 0.0005^B^	0.0184 ± 0.0005^B^	∞^X^	39.73 ± 1.15^Y^	37.78 ± 1.03^Y^
6	8.83	15,431.23	0.20 ± 0.07^α^	3.09 ± 0.11^β^	3.50 ± 0.13^γ^	0.0006 ± 0.0005^A^	0.0157 ± 0.0006^B^	0.0169 ± 0.0007^B^	1155.24 ± 11.65^X^	44.11 ± 1.69^Y^	41.02 ± 1.74^Y^
7	15.24	18,739.35	0^α^	3.24 ± 0.09^β^	3.71 ± 0.09^γ^	0^A^	0.0142 ± 0.0006^B^	0.0153 ± 0.0005^B^	∞^X^	48.97 ± 2.07^Y^	45.38 ± 1.56^Y^
8	15.94	22,199.11	0.20 ± 0.13^α^	3.00 ± 0.07^β^	3.66 ± 0.07^γ^	0.0002 ± 0.0001^A^	0.0129 ± 0.0005^B^	0.0138 ± 0.0005^B^	3465.22 ± 6.45^X^	53.88 ± 2.13^Y^	50.40 ± 1.96^Y^
9	7.57	23,842.39	0.12 ± 0.09^α^	3.08 ± 0.06^β^	3.98 ± 0.06^γ^	0.0002 ± 0.0001^A^	0.0120 ± 0.0007^B^	0.0126 ± 0.0005^B^	3465.22 ± 6.45^X^	57.97 ± 3.30^Y^	55.26 ± 1.94^Y^
10	14.93	27,083.19	0.20 ± 0.06^α^	2.79 ± 0.04^β^	4.08 ± 0.04^γ^	0.0005 ± 0.0004^A^	0.0106 ± 0.0006^B^	0.0118 ± 0.0004^B^	1386.78 ± 8.15^X^	65.56 ± 3.53^Y^	58.75 ± 2.22^Y^
11	12.61	29,819.60	0^α^	2.58 ± 0.08^β^	4.27 ± 0.07^γ^	0^A^	0.0094 ± 0.0003^B^	0.0112 ± 0.0003^C^	∞^X^	73.44 ± 2.11^Y^	62.05 ± 1.93^Z^
12	11.27	32,265.64	0^α^	2.42 ± 0.01^β^	4.61 ± 0.07^γ^	0^A^	0.0085 ± 0.0003^B^	0.0107 ± 0.0003^C^	∞^X^	82.07 ± 2.55^Y^	64.77 ± 1.95^Z^
13	8.80	34,175.72	0^α^	2.31 ± 0.01^β^	4.51 ± 0.03^γ^	0^A^	0.0081 ± 0.0003^B^	0.0100 ± 0.0003^C^	∞^X^	86.13 ± 3.09^Y^	69.49 ± 2.33^Z^
14	9.25	36,183.65	0^α^	2.15 ± 0.05^β^	4.61 ± 0.04^γ^	0^A^	0.0076 ± 0.0004^B^	0.0095 ± 0.0003^C^	∞^X^	91.92 ± 4.59^Y^	73.22 ± 2.26^Z^
15	7.66	37,846.13	0^α^	2.07 ± 0.01^β^	4.46 ± 0.02^γ^	0^A^	0.0070 ± 0.0003^B^	0.0089 ± 0.0003^C^	∞^X^	98.88 ± 3.75^Y^	78.20 ± 2.41^Z^
16	13.43	40,760.84	0^α^	1.86 ± 0.04^β^	4.18 ± 0.05^γ^	0^A^	0.0062 ± 0.0002^B^	0.0083 ± 0.0002^C^	∞^X^	112.41 ± 3.88^Y^	83.21 ± 2.46^Z^
17	14.25	43,854.07	0^α^	1.76 ± 0.02^β^	4.48 ± 0.06^γ^	0^A^	0.0060 ± 0.0002^B^	0.0082 ± 0.0002^C^	∞^X^	116.65 ± 4.75^Y^	85.08 ± 2.37^Z^
18	6.99	45,371.90	0^α^	1.68 ± 0.01^β^	4.55 ± 0.05^γ^	0^A^	0.0057 ± 0.0003^B^	0.0078 ± 0.0002^C^	∞^X^	122.87 ± 6.44^Y^	88.88 ± 2.53^Z^

Data were expressed as Mean ± SD (*n* = 3). αβγ, ABC and XYZ show significant differences among control and different concentrations of sodium bicarbonate treatments under the same cultivation day (one-way ANOVA; Tukey multiple comparison; *p* < 0.05)

^a^TPAR, received total PAR mole per square meter per day

^b^APARE, accumulated total PAR energy per square meter with day

^c^Control, culture without adding sodium bicarbonate (0.00 mol L^−1^)

^d^Treatment 1, culture adding 0.02 mol L^−1^ sodium bicarbonate

^e^Treatment 2, culture adding 0.10 mol L^−1^ sodium bicarbonate

^f^0, indicating that no photosynthetic efficiency or specific growth rate was found under specific cultivation day

^g^∞, indicating that doubling time tended to infinity under specific cultivation day

As compared to the control which showed no growth, the specific growth rates (*μ*) of cultures in Treatment 1 and Treatment 2 were comparable in the first 11 days (Table 1), recording a maximum *μ* on Day 5 (Treatment 1 = 0.0175 h^−1^; Treatment 2 = 0.0184 h^−1^) that was also significantly higher than *D. salina* cultured in an open pond bubbling with CO_2_ although lower than *Chlorella sorokiniana* cultivated in an outdoor cylindrical hanging bag PBR (Tables 1, 2) [24, 25]. Correspondingly, the shortest doubling time was achieved on Day 5 (Treatment 1 = 39.73 h^−1^; Treatment 2 = 37.78 h^−1^), respectively, which was also significantly shorter than those of most of reported PBRs listed in Table 2 (Table 1).

**Table 2:** Comparisons of rotating floating photobioreactor with other reported photobioreactor

Photobioreactor type	Location	Species used	Biomass productivity		Photosynthetic efficiency	Specific growth rate	Doubling time	References
			Areal	Volumetric	(PE)	(*μ*)	(*t*_d_)	
			(g m^−2^ day^−1^)	(g L^−1^ day^−1^)	(%)	(h^−1^)	(h)	
RFP	Outdoors	*D. tertiolecta*	3.10	0.06	4.61	0.0184	37.78	This study
Open raceway pond	Outdoors	*D. salina*	18.00	0.15	–	0.0069	100.43	[26]
Open raceway pond	Outdoors	*Nannochloropsis* sp.	14.00	0.08	1.50	0.0050	138.60	[30]
Closed horizontally tubular PBR	Outdoors	*D. salina*	2.00	0.06	2.00	0.0075	92.40	[16]
Closed horizontally tubular PBR	Outdoors	*Muriellopsis* sp.	40.00	–	4.40	0.0900	7.70	[30]
Closed horizontally tubular PBR	Outdoors	*Nannochloropsis* sp.	15.70	0.85	1.80	0.0142	48.80	[30]
Vertically annular column PBR	Outdoors	*T. suecica*	36.30	0.48	9.40	0.0136	51.12	[31]
Vertical tubular PBR	Outdoors	*Nannochloropsis* sp.	24.40	0.71	4.20	0.0167	41.50	[30]
Hanging bag PBR	Outdoors	*D. tertiolecta*	–	0.17	–	0.0108	64.17	[27]
Cylindrical hanging bag PBR	Outdoors	*Chlorella sorokiniana*	–	0.22	–	0.0521	13.30	[25]
Flat-panel airlift PBR	Indoors	*D. tertiolecta*	–	0.18	–	0.0242	28.64	[28]
Flat-panel PBR	Outdoors	*Nannochloropsis* sp.	27.50	1.20	3.80	0.0150	46.20	[30]
Green wall panel PBR	Outdoors	*Chlorella* sp.	11.23	0.60	2.80	–	–	[29]

– indicating that no related value was found

In contrast to the indoors data, however, the highest volumetric biomass productivity (0.06 g L^−1^ day^−1^) and *μ* (0.0184 h^−1^) of *D. tertiolecta* in RFP under Treatment 2 were lower than those (0.18 g L^−1^ day^−1^ and 0.0242 h^−1^, respectively) of *D. tertiolecta* BE003 cultured in indoors flat-panel airlift PBRs in constant light intensity (Table 2) [26]. Lower biomass productivity in varying light conditions in outdoor culture, despite higher total light energy received over the day, would need to be investigated.

By means of rotating floating photobioreactor, the photosynthetic efficiencies of *D. tertiolecta* under different sodium bicarbonate levels were fully determined in this study. Other than the control which photosynthetic efficiencies during all the cultivation days were very low as there was no increase in biomass, photosynthetic efficiencies of Treatments 1 and 2 indicated their highest values of 3.24 % on Day 7 and 4.61 % on Day 14, respectively (Table 1). Before Day 5, the photosynthetic efficiencies of both treatments were almost the same. It was obvious that the photosynthetic efficiency under Treatment 2 remained stable above 4 % after Day 10, whereas the photosynthetic efficiency under Treatment 1 decreased towards the end of the experiment (Table 1).

In terms of photosynthetic efficiency, there were two definitions, one based on biomass yield on light energy absorbed (gram protein produced per mol of photons absorbed), and the second based on energy stored in biomass per unit of light energy absorbed, which was adopted in this study [18, 27]. In comparison with other similar studies where microalgae were cultured in outdoor land-based PBRs, *D. tertiolecta* cultured in RFP achieved higher PE value (4.61 %) than that (2 %) of *D. salina* and that (4.4 %) of *Muriellopsis* sp. cultivated in closed horizontally tubular PBRs as well as those of *Nannochloropsis* sp. cultured in four conventional PBRs including open raceway pond, horizontal tubular PBR, vertical tubular PBR and flat-panel PBR along with *Chlorella* sp. in a green wall panel PBR (Tables 1, 2) [16, 28–30]. However, the PE achieved in this study is lower than that (9.4 %) of *Tetraselmis suecica* cultured in a vertically annular columns [31]. It has been recognized that optimizing PBR placement might help decrease photosaturation phenomenon encountered at noon in outdoor microalgal cultures [11, 32]. In a PBR placed at an angle towards the sun, sunlight could spread over a larger bioreactor surface area as compared to a PBR placed vertically or horizontally, resulting in each microalgal cells receiving lower light intensities and achieving higher PE [32]. Looking into our RFP design, the six culture barrels were placed parallel to the water level and roller of the RFP (Fig. 1a). The design of the RFP could be further refined to overcome the orientation issue.

**Fig. 3:**
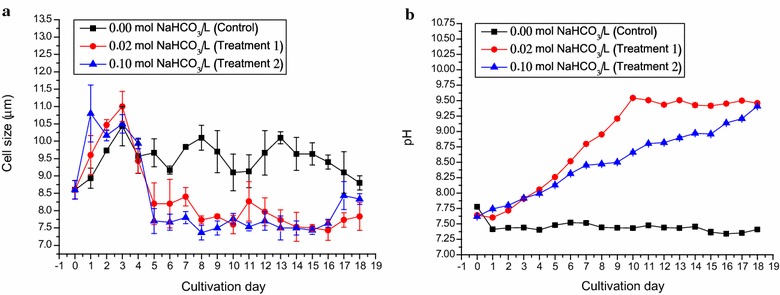
The cell size and pH value of *Dunaliella tertiolecta* cultured outdoors under different sodium bicarbonate concentrations in ATCC-1174 DA medium using rotating floating photobioreactor. **a** The changes in cell sizes of *D. tertiolecta* under 0.02 mol L^−1^ NaHCO_3_ (*filled red circle*) and 0.10 mol L^−1^ NaHCO_3_ (*filled blue triangle*) as compared to the control, 0.00 mol L^−1^ NaHCO_3_ (*filled black cube*). **b** The changes in pH values of *D. tertiolecta* culture under cultivation conditions mentioned above. Data were expressed as mean ± standard deviation of three independent experiments (*n* = 3)

The cell size of *D. tertiolecta* and pH of the culture were monitored daily in this study (Fig. 3). Initially, the cell size of *D. tertiolecta* inoculum was about 8.60 µm. In the first 3 days, cell sizes under all three conditions increased to about 11 µm. The cell size under control condition maintained unchanged, ranging from 9 to 10 µm throughout the whole cultivation. In Treatments 1 and 2 the cell size decreased significantly (*p* < 0.05) to approximately 7–8.50 µm (Fig. 3a), which is an indication of fast growth.

The pH under these three cultivations demonstrated different trends. The original medium pH was 7.62 to 7.78. The pH of the control cultures dropped to 7.35–7.50 after the experiment. The pH under Treatment 1 gradually raised to 9.54 on Day 10 and stayed steady till the end of the study. The pH of Treatment 2 cultures went up to 9.41 on Day 18 (Fig. 3b). According to the principle of carbonate equilibria, it was noted that the utilization and removal of bicarbonates from culture by *D. tertiolecta* resulted in the increases of pH in culture that might further lead to the decrease of bicarbonate (${\text{HCO}}_{ 3}^{ - }$) level by shifting to carbonate (${\text{CO}}_{ 3}^{2 - }$) which was the inorganic form that could not be used and absorbed by photosynthetic microalgae [33]. This might be another explanation for lower than the theoretical biomass concentration that could be reached in high carbonate medium (Fig. 2d). In addition, it was intriguing that culture pH value under Treatment 1 was significantly (*p* < 0.05) higher than that under Treatment 2 throughout most of the time of cultivation (Fig. 3b). The explanation could be due to the fact that dissolved bicarbonate could act as the buffer solution to maintain pH in the high carbonate medium. Nevertheless, the pH in this study was within the optimum growth range of the alga, thus changes in biomass productivity should not be an effect of culture pH.

**Fig. 4:**
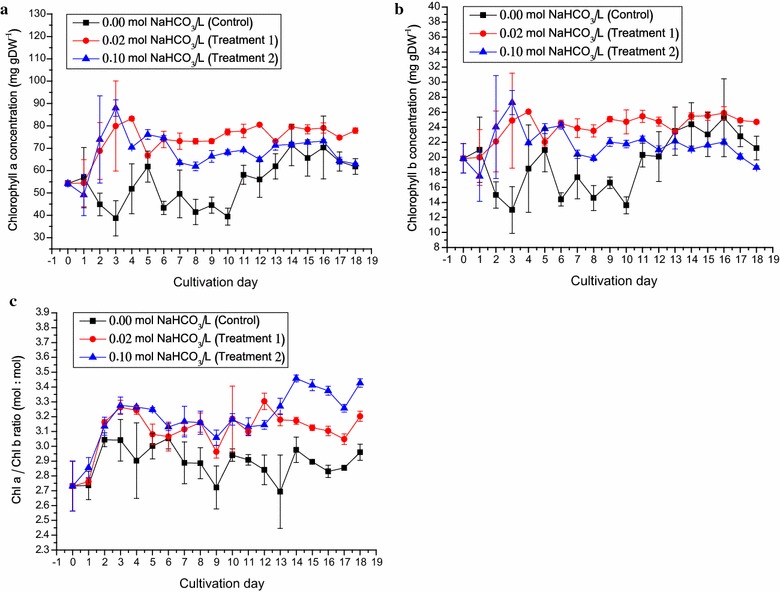
The concentrations of chlorophyll *a* and *b* as well as chlorophyll *a*/*b* ratio of *Dunaliella tertiolecta* cultured outdoors under different sodium bicarbonate concentrations in ATCC-1174 DA medium using rotating floating photobioreactor. **a** The changes in chlorophyll *a* levels of *D. tertiolecta* under 0.02 mol L^−1^ NaHCO_3_ (*filled red circle*) and 0.10 mol L^−1^ NaHCO_3_ (*filled blue triangle*) as compared to the control, 0.00 mol L^−1^ NaHCO_3_ (*filled black cube*). **b** The changes in chlorophyll *b* levels of *D. tertiolecta* culture under cultivation conditions mentioned above. **c** The changes in chlorophyll *a*/*b* ratio of *D. tertiolecta* culture under cultivation conditions mentioned above. Data were expressed as mean ± standard deviation of three independent experiments (*n* = 3)

The original chlorophyll *a* and *b* concentrations of *D. tertiolecta* inoculum were 54.23 and 19.84 mg gDW^−1^, respectively (Fig. 4a, b). The chlorophyll *a* content of the control cultures increased from 40 to 70 mg gDW^−1^. The chlorophyll *a* in Treatment 1 cultures remained between 70 and 80 mg gDW^−1^. The chlorophyll *a* of the Treatment 2 cultures declined after Day 6, reaching that of the control cultures (Fig. 4a).

The chlorophyll *b* level of cultures in the three treatment conditions varied after Day 13 (Fig. 4b). It is interesting to note that the levels of both chlorophyll *a* and *b* under their respective culture conditions followed roughly the same trends (Fig. 4a, b).

The chlorophyll *a*/*b* ratio was an indication of the photosynthetic antenna cross section. The initial chlorophyll *a*/*b* ratio was 2.73, and varied only between 2.69 and 3.05 under control condition (Fig. 4c). The ratios under Treatments 1 and 2 remained relatively similar throughout the experiment, but significantly (*p* < 0.05) higher than that of control in most of the time (Fig. 4c).

These were in agreement with related studies that addition of sodium bicarbonate or CO_2_ increased Chl *a* and Chl *b* concentrations in microalgal cells [34–36].

**Fig. 5:**
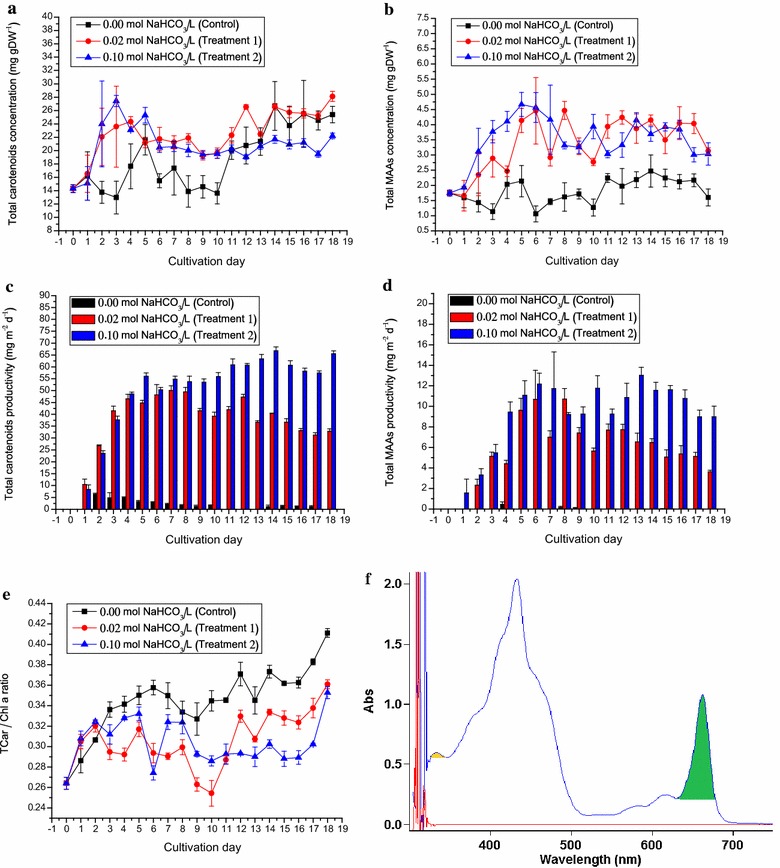
The total carotenoids and total MAAs of *Dunaliella tertiolecta* cultured outdoors under different sodium bicarbonate concentrations in ATCC-1174 DA medium using rotating floating photobioreactor. **a** The changes in total carotenoid concentrations of *D. tertiolecta* under 0.02 mol L^−1^ NaHCO_3_ (*filled red circle*) and 0.10 mol L^−1^ NaHCO_3_ (*filled blue triangle*) as compared to the control, 0.00 mol L^−1^ NaHCO_3_ (*filled black cube*). **b** The changes in total MAA concentrations of *D. tertiolecta* culture under cultivation conditions mentioned in Fig. 5a. **c** The changes in total carotenoid productivity (g m^−2^ day^−1^) of *D. tertiolecta* culture under 0.02 mol L^−1^ NaHCO_3_ (*red bar*) and 0.10 mol L^−1^ NaHCO_3_ (*blue bar*) as compared to the control, 0.00 mol L^−1^ NaHCO_3_ (*black bar*). **d** The changes in total MAA productivity (g m^−2^ day^−1^) of *D. tertiolecta* culture under cultivation conditions mentioned in Fig. 5c. **e** The changes in total carotenoid/chlorophyll *a* ratio of *D. tertiolecta* culture under cultivation conditions mentioned in Fig. 5a. **f** The scanning spectrograph of *D. tertiolecta* from 300 to 750 nm; the yellow peak (319–350 nm) and green peak (630–679 nm) stand for the absorption peaks of total MAAs and chlorophyll *a*, respectively. The negative values of productivities calculated were all shown as 0 g m^−2^ day^−1^. Data were expressed as mean ± standard deviation of three independent experiments (*n* = 3)

The original total carotenoid concentration of *D. tertiolecta* inoculum was 14.33 mg gDW^−1^ (Fig. 5a). Before Day 10, the total carotenoids under Treatments 1 and 2 appeared to accumulate in the *D. tertiolecta* cells as compared to that in the control cultures. At the later stage of study, the control cultures accumulated total carotenoids to the level of Treatment 1, and significantly (*p* < 0.05) higher than Treatment 2 which remained stable between 19.42 and 22.21 mg gDW^−1^ after Day 6 (Fig. 5a).

The total carotenoid/chlorophyll *a* ratio in Treatment 2 was higher than that in Treatment 1 at the early stage prior to Day 11, but lower at the later stage, but both were lower than that the control throughout the study (Fig. 5e).

In the control condition, production of carotenoids was not induced. The total carotenoid yield of Treatment 1 was 50.05 mg m^−2^ day^−1^, attending the maximum on Day 7 and gradually decreased to around 30 mg m^−2^ day^−1^ by the end of the study. In Treatment 2, the carotenoid productivity increased during the study to reach at value of 66.83 mg m^−2^ day^−1^ on Day 14 (Fig. 5c).

The total MAA level in *D. tertiolecta* originally detected was 1.75 mg gDW^−1^ (Fig. 5b, f). Under control condition where no carbon sources were supplied, the total MAA concentrations ranged only from 1.06 to 2.47 mg gDW^−1^. In Treatment 1, the MAAs reached 4.47 mg gDW^−1^ on Day 8, whereas in Treatment 2, the MAAs reached 4.67 mg gDW^−1^ on Day 5 (Fig. 5b).

The MAA productivities in Treatments 1 and 2 were similar at the early stage prior to Day 8. Subsequently, total MAA productivity in Treatment 2 (13.04 mg m^−2^ day^−1^ on Day 13) exceeded that of Treatment 1 (10.70 mg m^−2^ day^−1^ on Day 8) at the late stage (Fig. 5d).

Significant (*p* < 0.05) increases in total carotenoid and MAA levels of *D. tertiolecta* under Treatment 1 and 2 were found as compared to that in the control and at Day 0 (Fig. 5a, b). MAAs are amino acids or reduced amino acid derivatives having 2 cyclic units including an aminocyclohexenone (Mycosporines) and an aminocyclohexenimide with absorption maxima ranging from 310 to 360 nm in the UV region [37]. Thus, it might suggest that the accumulations of total carotenoids and MAAs in the *D. tertiolecta* cells under both bicarbonate enriched conditions might be protective mechanisms for screening the harmful irradiance from the growing cultures [38], as the RFP exposed the algal cells to high light intensity more frequently.

**Fig. 6:**
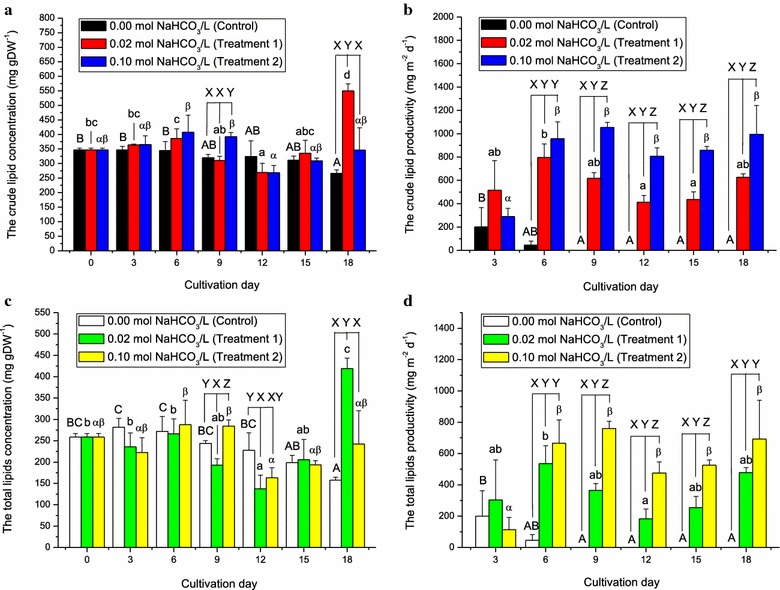
The crude lipids and total lipids (minus chlorophyll *a* and *b* and total carotenoids) of *Dunaliella tertiolecta* cultured outdoors under different sodium bicarbonate concentrations in ATCC-1174 DA medium using rotating floating photobioreactor. **a** The changes in crude lipid concentrations of *D. tertiolecta* culture under 0.02 mol L^−1^ NaHCO_3_ (*red bar*) and 0.10 mol L^−1^ NaHCO_3_ (*blue bar*) as compared to the control, 0.00 mol L^−1^ NaHCO_3_ (*black bar*). **b** The changes in crude lipid productivity (g m^−2^ day^−1^) of *D. tertiolecta* culture under cultivation conditions as mentioned in **a**. **c** The changes in total lipid concentration of *D. tertiolecta* culture under 0.02 mol L^−1^ NaHCO_3_ (*green bar*) and 0.10 mol L^−1^ NaHCO_3_ (*yellow bar*) as compared to the control, 0.00 mol L^−1^ NaHCO_3_ (*white bar*). **d** The changes in total lipid productivity (g m^−2^ day^−1^) of *D. tertiolecta* culture under cultivation conditions mentioned in c. The negative values of productivities calculated in control condition were all shown as 0 g m^−2^ day^−1^. Data were expressed as mean ± standard deviation of three independent experiments (*n* = 3). ABC, abcd, and αβ indicated significant differences among different cultivation days under the control, Treatment 1, and Treatment 2, respectively. XYZ showed significant differences among control and different concentrations of sodium bicarbonate under the same cultivation day (one-way ANOVA; Tukey multiple comparison; *p* < 0.05)

The crude lipids and total lipids were determined every three days. The original levels of crude and total lipids found in *D. tertiolecta* cells were 346.83 and 258.44 mg gDW^−1^, respectively (Fig. 6a, c). The level of crude lipids of *D. tertiolecta* under control condition gradually decreased as compared to the initial level. In Treatment 1, there was significant (*p* < 0.05) accumulation of lipids reaching 418.67 mg gDW^−1^ on Day 18 (Fig. 6c).

The highest production rate of crude lipids of 1052.96 mg m^−2^ day^−1^ was observed at mid-term of cultivation on Day 9 under Treatment 2. The crude lipid productivity under Treatment 1 only reached 795.41 mg m^−2^ day^−1^ on Day 6 (Fig. 6b). The total lipid productivities under all the cultivation conditions followed the trends of crude lipids, ranging from 0 to 760.28 mg m^−2^ day^−1^ (Fig. 6b, d).

Recent publications have demonstrated positive relationship between bicarbonate concentrations and lipid accumulation in microalgae, such as chlorophyte *Scenedesmus* sp., marine diatom *Phaeodactylum tricornutum* strain Pt-1, *Tetraselmis suecica*, *Nannochloropsis salina*, and *Chaetoceros gracilis* [35, 36, 39]. In our case, the significant (*p* < 0.05) accumulation of lipids in RFP were found in Treatment 2 at Day 9 and under Treatment 1 at Day 18 (Fig. 6a, c). This could be attributed to the supply of carbon in the form for dissolved bicarbonate and frequent exposure to higher light intensity.

### Comparison on solar energy capture capability of rotating floating photobioreactor and conventional photobioreactors

Calculation on solar energy capture capability of RFP and conventional PBRs with different placement of comparable foot print is shown in Table 3.

**Table 3:** Solar energy capture capabilities of rotating floating photobioreactor and conventional photobioreactors under the same foot print

Photobioreactor type	Thickness/diameter (m)	Total length^a^/height (m)	Width (m)	Effective volume^b^ (L)	Expected productivity (g m^−2^ day^−1^)	Captured solar energy^c^ (kJ m^−2^ day^−1^)
RFP	0.140	2.280	0.470	18.000	3.10	62.0
Vertical column/tubular PBR	0.470	0.470	–	22.480	3.87	77.4
Horizontal column/tubular PBR	0.035	9.620	–	9.256	1.59	31.8
Inclined column/tubular PBR	0.030	13.149	–	9.294	1.60	32.0
Vertical plane PBR	0.070	0.740	0.470	24.346	4.19	83.8
Flat plane PBR	0.035	0.740	0.470	12.173	2.10	42.0
Inclined plane PBR	0.030	0.877	0.470	12.366	2.13	42.6

The configuration of compared conventional photobioreactors should be within the space usage of current RFP as 0.74 m length, 0.47 m width, and 0.47 m height

^a^The total lengths of column/tube that can be arranged in an area of 0.74 m length and 0.47 m width

^b^The effective volumes of photobioreactors which allow solar lights to penetrate 0.035 m depth of culture

^c^Under normal growth condition without stress, 1 g of algal biomass contains 20 kJ energy [18]

It was obvious from Table 3 that the solar energy capture capability of RFP was superior to those of the horizontal and inclined PBRs, but inferior to those of the vertical ones, indicating that appropriate placement of PBR might help capture more solar energy under the same scale and the modification on RFP by designing its barrels vertical to the axle might be helpful. This observation is also consistent with the fact that vertically placed PBR showed higher PE as compared to those of the horizontally placed ones [16, 28, 31]. In addition, although RFP is also a horizontal column/tubular PBR, it has an advantage over the conventional horizontally placed column/tubular PBRs for its ability to capture almost double the solar energy over the same foot print (Table 3).

### Required flowing water power and determining factors for successful performance of rotating floating photobioreactor

In order to facilitate the rotation of RFP, the original power provided by the flowing water is required to conquer not only the force of friction generated by the net force of RFP axle (the total RFP weight minus the buoyancy) pressing the shafts but also the effect of water resistance that may hinder its motion while rotating.

In this study, the total weight of RFP including the culture medium was 25 kg. As the volume of RFP underwater ($V_{\text{underwater}}$) was 0.02059 m^3^ which included the underwater parts of containers, axis and paddles, the buoyancy (*F*_buoyancy_) received by the RFP at 25 °C was thus,

11 $$\begin{aligned}F_{\text{buoyancy}} &= \rho_{{{\text{water}} \, (25\,^\circ {\text{C}})}} V_{\text{underwater}} g \\ &= 996.8 \times 0.02059 \times 9.8 = 201.14\,{\text{N}} ,\end{aligned}$$

in which $\rho_{{{\text{water}}(25\,^\circ {\text{C}})}}$ is the density of water at 25 °C.

Therefore, the net force ($N_{\text{net}}$) pressing the shafts will be

12 $$N_{\text{net}} = G_{\text{RFP}} - F_{\text{buoyancy}} = 25 \times 9.8 - 201.14 = 43.86\,{\text{N}} .$$

Because both of axle and shafts were made of PVC in this study and the static friction coefficient ($\mu_{\text{PVC}}$) of PVC is 0.5, the force ($F_{\text{anti-friction}}$) to conquer the friction of net force on the shafts could therefore be represented as

13 $$F_{\text{anti-friction}} = \mu_{\text{PVC}} N_{\text{net}} = 0.5 \times 43.86 = 21.93\,{\text{N}} .$$

On the other hand, the force to conquer water resistance encountered by RFP is also of significance. The rotation rate of RFP was 3 s of per cycle, which led to 0.492 m s^−1^ of the maximal lineal velocity on the RFP circumference and decreasing lineal velocity along the paddle till zero at the center of axle, the water resistance received at the different positions of the paddle increased progressively from the axle center to the circumference of RFP according to the formula for water resistance of underwater object:

14 $$F_{\text{anti-resistance}} = \frac{1}{2}\rho_{{{\text{water}} \, (25\,^\circ {\text{C}})}} S_{\text{underwater}} v_{\text{RFP}}^{2} ,$$

in which $F_{\text{anti-resistance}}$ is the force required by underwater paddle to conquer water resistance at certain position, $S_{\text{underwater}}$ represents the underwater cross section of paddle, and $v_{\text{RFP}}$ stands for the lineal velocity at the resisted position of paddle.

As a result, the water resistance received at a certain position of the paddle is

$$\begin{aligned} {\text{d}}F_{\text{anti-resistance}} = \frac{1}{2}\rho_{{{\text{water}} \, (25\,^\circ {\text{C}})}} v_{\text{RFP}}^{2} dS_{\text{underwater}} \\ \quad = \frac{1}{2}\rho_{{{\text{water}} \, \left( {25\,^\circ {\text{C}}} \right)}} \left( {\frac{0.492}{0.235}x} \right)^{2} L{\text{d}}x \end{aligned}$$

15 $$\begin{aligned} &= \frac{1}{2}\rho_{{{\text{water}} \, \left( {25\,^\circ {\text{C}}} \right)}} \left( {\frac{0.492}{0.235}x} \right)^{2} L{\text{d}}x \\ &= 983.07x^{2} {\text{d}}x \end{aligned}$$

in which $x$ stands for the direction along the paddle, the distance from the axle center to RFP circumference is 0.235 m, and $L$ is the width of paddle which is 0.45 m in this study.

The integration total accumulation of water resistance on a paddle could therefore be calculated from the axle center to RFP circumference (0–0.235 m) based on Eq. (15), and is given as

16 $$\begin{aligned} F_{\text{anti-resistance}} &= \mathop \int \limits_{0}^{0.235} 983.07x^{2} {\text{d}}x \\ \quad &= \left[ {\frac{983.07}{3}x^{3} } \right]_{0}^{0.235} = 4.25\,{\text{N}}. \end{aligned}$$

As 3 paddles of RFP were normally kept under the water at any time of rotation, the total force for overcoming water resistance by the current RFP is therefore given as

17 $$\begin{aligned} F_{\text{total anti-resistance}} &= 3 \times F_{\text{anti-resistance}} \\ &= 3 \times 4.25 = 12.75\,{\text{N}} . \end{aligned}$$

So, the minimal force ($F_{\hbox{min} }$) provided by the power from flowing water to support and rotate 25 kg of RFP in this study was

18 $$\begin{aligned} F_{\hbox{min} } &= F_{\text{anti-friction}} + F_{\text{total anti-resistance}} \\ &= 21.93 + 12.75 = 34.68\,{\text{N}} , \end{aligned}$$

which meant that at least 3.54 kg (34.68 N) of water force was required to push 25 kg of currently designed RFP to rotate.

In order to provide at least 34.68 N of force to 0.106 m^2^ (0.235 m × 0.45 m) of the paddle to facilitate rotation, the power (*E*) obtained from the flowing water of a flow rate ($v_{\text{water}}$) could be estimated as follows:

19 $$\begin{aligned} E &= F_{\rm{min} } \cdot L = \frac{1}{2}m_{\text{water}} v^{2} \\ \quad &= \frac{1}{2}\rho_{{{\text{water}} \, \left( {25\,^\circ {\text{C}}} \right)}} V_{\text{water}} v_{\text{water}}^{2} \\ \quad &= \frac{1}{2}\rho_{{{\text{water}} \, \left( {25\,^\circ {\text{C}}} \right)}} \cdot S \cdot L \cdot v_{\text{water}}^{2} , \end{aligned}$$

20 $$F_{\rm{min} } = \frac{1}{2}\rho_{{{\text{water}} \, \left( {25\,^\circ {\text{C}}} \right)}} \cdot S \cdot v_{\text{water}}^{2} ,$$

in which $m_{\text{water}}$ and $V_{\text{water}}$ stand for the mass and volume of the performing water on RFP, respectively.

As a result,

21 $$\begin{aligned} v_{\text{water}} &= \sqrt {\frac{{2F_{\hbox{min} } }}{{\rho_{{{\text{water}}\left( {25\,^\circ {\text{C}}} \right)}} \cdot S}}} \\ &= \sqrt {\frac{2 \times 34.68}{996.8 \times 0.106}} = 0.810\,{\text{m}}\,{\text{s}}^{ - 1} . \end{aligned}$$

In other words, the flow rate of the flowing water in the flowing water should be above 0.810 m s^−1^ in order to achieve the rotation motion of RFP in this study. The flow rate of water in the raceway pond in this study was 0.831 m s^−1^ and was able to move the RFP as predicted by the estimation above.

From the above, it is clear that the essential conditions to successfully perform RFP in flowing water are subjected to the energy contained in the flowing water determined by the lineal flow rate and how much flowing water power was captured and absorbed by RFP through ascertaining the cross-section areas of the paddles. On the other hand, the configuration and total weight of RFP would affect the buoyancy and net force of axle pressing the shafts and further influence the change of friction; moreover, the static friction coefficient between axle and shaft is also related to the event. Based on the flow rate of a water body, the size and configuration for a determined volume of algal culture could be constructed accordingly.

### Using CO_2_ as alternative carbon source in the rotating floating photobioreactor for more economical cultivation

CO_2_-enriched air could be used as the carbon source for RFP for economical reason as sodium bicarbonate currently used is of a higher cost. Alternatively, pure CO_2_ may also be supplied through permeable membrane balloon because the barrels of RFP contained only half of their capacity to increase buoyancy.

The balloon is recommended to be made of the gas permeable polydimethylsiloxane (PDMS) membrane. PDMS demonstrates high intrinsic flux (3190 Barrer) of CO_2_ permeability [40]. Thus, pure CO_2_ from industrial waste gas, such as that from breweries, could be used directly.

### Theoretical improvement of rotating floating photobioreactor in scale up for mass cultivation of microalgae

It is worth mentioning that the currently studied RFP demonstrated lower biomass productivity as compared to some of the PBRs listed in Table 2. It remains that the current RFP is only a small-scale conceptual model.

In RFP, its areal productivity is a function of radius, as a doubling in the radius of the culture barrel while keeping its length, resulted in a doubling in its water footprint, but four times increased in the culture volume. Thus, the areal productivity of a RFP increased exponentially with increasing radius of the culture vessels.

To circumvent the effect of self-shading and intermittent exposure to light in scale up, static mixers could be installed inside the culture barrels.

### The recommended sites for installation of rotating floating photobioreactor

**Fig. 7:**
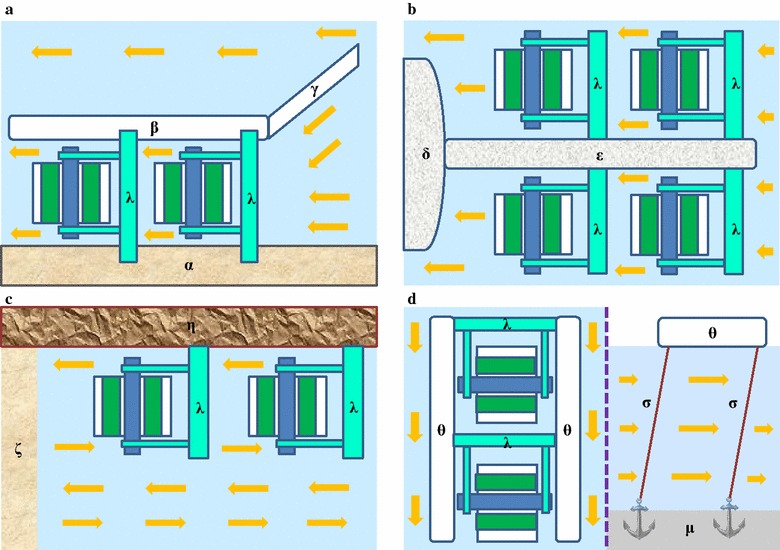
Several recommended sites for installation of RFR. **a** Along a riverbank. **b** Protruding into a river. **c** At a seashore. **d** In a river. *α* riverbank, *β* auxiliary bank, *γ* baffle bank, *δ* pier, *ε* extension dam of a pier, *ζ* seashore, *η* dam stretched into the sea, *θ* long boat, *λ* stand bar, *μ* riverbed, *σ* steel cable. *Yellow arrows* indicate the directions of flowing water

Theoretically, all flowing water bodies are suitable for installation of RFP. Riverbank is an ideal place for operating RFP and the auxiliary bank could be constructed to form an artificial canal. The baffle bank is recommended for collecting more flowing water (more energy) in the canal to accelerate the water flow (Fig. 7a). The extension dam of a pier may be the second desirable location for RFP performance (Fig. 7b).

The man-made dam located at seashore could also be used for installation of RFP system. As the waves at the seashore are normally a reciprocating one, the rotation direction of RFP may thus be rhythmically reciprocating subjected to the frequency and direction of waves (Fig. 7c). In any flowing water area, the RFP could be set up between two parallel long boat stations by anchors (Fig. 7d).

### Economical evaluation

The economic viability of RFP is excellent as compared to those of the traditional photobioreactors, at least, in the aspects of culture mixing and temperature control costs [12, 41]. For the most cost-effective horizontal tubular photobioreactor, the total cost for producing a kg dry weight (DW) of microalgae was estimated at 4.15 €, with a mixing cost of 1.27 €, which accounted for 31 % of the total cost [12]. The RFP reported here does not have a mixing cost, thus dropping the production cost by 31 %. Another economic advantage of RFP is saving on the cost for temperature control. It was reported that the cost of construction of a cooling system for an industrial-size flat plate glass reactor might account for 34 % of total investment cost. Moreover, the cooling cost amounted to 30 % of the total production cost in the photobioreactor [41].

## Conclusions

The RFP developed are recommended for installation in flowing brook, river, and channel with stable unidirectional waves, taking advantage of free natural hydraulic energy. Water bodies near hydrojunction and hydraulic power plant are highly suitable for large-scale heavier RFP. The natural reciprocating waves at seashore and in ocean are also compatible for RFP because of the reciprocating rotation. In outdoor conditions, the RFP achieved higher *D. tertiolecta* biomass productivity as well as higher productivity of carotenoids, MAAs, and lipids, as compared to many of those reported in the literature.

## Nomenclature

### List of symbols

*A*_Chl*a*_: the peak area in the range of 630–680 nm
*A*_MAAs_: the total peak area in the range of 319–350 nm
*E*_B_: the free energy included in biomass (kJ)
*E*_L_: the free energy included in light energy (kJ)
*M*_0_: the mass (g m^−2^) measured on day 0
*m*_1_: the weight of glass microfiber filter before used
*m*_2_: the weight of dried filter with sample/extracted crude lipids
*M*_*n*_: the mass (g m^−2^) measured on day *n*
*N*_0_: the cell numbers per mL of culture measured on day 0
*N*_*n*_: the cell numbers per mL of culture measured on day *n*
*t*_d_: doubling time (h)

### Greek symbols

*μ*: specific growth rate (h^−1^)

### Subscripts

B: biomass
Chl*a*: chlorophyll *a*
d: doubling
L: light energy
*n*: the time of cultivation (days)

## Abbreviations

ATCC: American Type Culture Collection at Manassas
DW: dry weight (g L^−1^)
MAAs: mycosporine-like amino acids
PAR: photosynthetically active radiation
PE: photosynthetic efficiency
PET: polyethylene terephthalate
PVC: polyvinyl chloride
RFP: rotating floating photobioreactor
UTEX: The University of Texas at Austin
